# Staff Recall Travel Time for ST Elevation Myocardial Infarction Impacted by Traffic Congestion and Distance: A Digitally Integrated Map Software Study

**DOI:** 10.3389/fcvm.2017.00089

**Published:** 2018-01-08

**Authors:** Justin Cole, Richard Beare, Thanh G. Phan, Velandai Srikanth, Andrew MacIsaac, Christianne Tan, David Tong, Susan Yee, Jesslyn Ho, Jamie Layland

**Affiliations:** ^1^Peninsula Health Heart Service, Frankston, VIC, Australia; ^2^Peninsula Clinical School, Monash University, Melbourne, VIC, Australia; ^3^Developmental Imaging, Murdoch Children’s Research Institute, Parkville, VIC, Australia; ^4^School of Clinical Sciences, Monash Health, Monash University, Melbourne, VIC, Australia; ^5^Department of Cardiology, St Vincent’s Hospital, Melbourne, VIC, Australia; ^6^School of Medicine, Monash University, Melbourne, VIC, Australia

**Keywords:** ST elevation myocardial infarction, time to percutaneous coronary intervention, ST elevation myocardial infarction protocol, staff travel time, catheterization laboratory activation

## Abstract

**Background:**

Recent evidence suggests hospitals fail to meet guideline specified time to percutaneous coronary intervention (PCI) for a proportion of ST elevation myocardial infarction (STEMI) presentations. Implicit in achieving this time is the rapid assembly of crucial catheter laboratory staff. As a proof-of-concept, we set out to create regional maps that graphically show the impact of traffic congestion and distance to destination on staff recall travel times for STEMI, thereby producing a resource that could be used by staff to improve reperfusion time for STEMI.

**Methods:**

Travel times for staff recalled to one inner and one outer metropolitan hospital at midnight, 6 p.m., and 7 a.m. were estimated using Google Maps Application Programming Interface. Computer modeling predictions were overlaid on metropolitan maps showing color coded staff recall travel times for STEMI, occurring within non-peak and peak hour traffic congestion times.

**Results:**

Inner metropolitan hospital staff recall travel times were more affected by traffic congestion compared with outer metropolitan times, and the latter was more affected by distance. The estimated mean travel times to hospital during peak hour were greater than midnight travel times by 13.4 min to the inner and 6.0 min to the outer metropolitan hospital at 6 p.m. (*p* < 0.001). At 7 a.m., the mean difference was 9.5 min to the inner and 3.6 min to the outer metropolitan hospital (*p* < 0.001). Only 45% of inner metropolitan staff were predicted to arrive within 30 min at 6 p.m. compared with 100% at midnight (*p* < 0.001), and 56% of outer metropolitan staff at 6 p.m. (*p* = 0.021).

**Conclusion:**

Our results show that integration of map software with traffic congestion data, distance to destination and travel time can predict optimal residence of staff when on-call for PCI.

## Introduction

Despite major improvements in the timely treatment of acute myocardial infarction, recent evidence suggests that guideline specified time to percutaneous coronary intervention (PCI) for a proportion ST elevation myocardial infarction (STEMI) presentations is not being met (1, 2). Ideal first medical call to device time of ≤90 min is a Class: 1/level: B recommendation in the ACCF/AHA guideline for the Management of STEMI (3). Multiple approaches such as ambulance pre-notification of a STEMI patient to a hospital emergency department and cardiology unit combined with hospital STEMI-code protocols to activate and mobilize crucial staff, are known to reduce this time (4, 5).

Thus far, evidence has consistently demonstrated that time-to-reperfusion has an impact on infarct size, left ventricular function and ultimately mortality (6–9). There are several factors that contribute to this time and these include door-to-balloon time as well as broader “system-delays,” that is, the delay between first medical contact and reperfusion therapy. This often overlooked factor, can be significantly affected by the environment outside the hospital with potential impact on the timely treatment of patients. System-delay has been shown to be an important predictor of patient outcomes, and an indicator of quality of care (10), yet there remains a void in approaches specifically addressing this issue.

Out-of-hours, catheter laboratory staff recall travel times realistically form an important component of system-delay and to date, this variable has not been investigated. In this article, we introduce methods for objectively estimating staff travel times and perform a proof-of-concept study comparing these times between two metropolitan settings under different traffic conditions. We hypothesized that recall staff travel times to an inner metropolitan hospital would be affected by traffic congestion to a much greater degree than to an outer metropolitan hospital. We also predicted that a proportion of staff who reside in locations most affected by traffic would not be able to return to hospital during peak traffic conditions, within a target time frame of 30 min following catheter laboratory activation—a time frame set out in the Mayo Clinic STEMI protocol (5). Using novel simulation technologies, we set out to create regional maps that graphically show the impact of traffic congestion on staff recall travel times, thereby producing a resource that could be used by staff to improve reperfusion time for STEMI.

## Materials and Methods

### PCI Centers

Two high volume PCI centers were selected in Melbourne, the capital city of the state of Victoria in Australia, one in an inner metropolitan (~2 km from Melbourne City center) and the other in an outer metropolitan setting (~60 km from city center).

### Addresses

Known staff home postcodes were selected, however true addresses were not used to protect the anonymity of staff. To predict staff recall travel times to the hospital in question, we first randomly generated ten addresses for each known staff home postcode, using Google Maps Application Programming Interface (API).^1^ Utilization of multiple addresses provides an estimate of within-postcode travel time variability. Postcode boundaries were obtained from the Victorian government.^2^

### Time and Distance Estimation

To generate staff recall travel times and travel distance, we used the ggmap (11) interface to the Google Maps “Distance” API. The ggmap (11) package was modified in-house to specify the departure time from each address and a traffic model based on the time of travel, so that varying traffic conditions could be taken into account. Google Maps API uses both historical and live traffic data to estimate travel time at different times of the day. Three traffic model predictions: best guess, optimistic or pessimistic can be used. The default setting “best guess” is the best estimate of travel time given what is known about both historical traffic conditions and live traffic. Pessimistic may be longer than the actual travel time on most days, although occasional days with particularly bad traffic conditions may exceed this value. Optimistic should be shorter than the actual travel time on most days, although occasional days with particularly good traffic conditions may be faster than this value. The pessimistic traffic model was used to estimate staff recall travel times in this study.

### Recall Times

Selected recall times were midnight, 6 p.m., and 7 a.m., to represent recall outside the normal working hours of 8:30 a.m. to 4:30 p.m. Times of 6 p.m. and 7 a.m. were chosen to highlight the effect of traffic congestion based on AusRoads (12) (the peak organization of Australasian road transport and traffic agencies) predictions of maximum traffic congestion, and midnight was used for comparison because traffic was negligible at this time. Traffic congestion is defined as the increase in time traveled (actual travel time − nominal travel time). Recurrent peak hour traffic congestion is caused by both demand and supply imbalance (number of vehicles on road, given road capacity) and weekday/weekend effects (factors that systemically vary between weekdays and weekends) (12). Google Maps API will only provide predicted travel times for a selected date in the future, thus we selected Wednesday August 16, 2017, a mid-week day (thereby negating the weekend effect) not affected by public holidays or school holidays.

### Visualization

R statistical environment (13) was used to interact with the data generated by Google Maps API automated queries. Postcode boundaries were first overlaid on a state-wide map of Victoria, Australia. Staff addresses were then plotted on the map and further color and size coded to represent travel time.

### Statistical Analysis

Peak hour travel times were compared to midnight travel times using multilevel mixed effects models to account for paired data (same source and destination addresses at different times of travel) and correlated measures (different addresses within the same postcodes). Similar models, without paired data, were used to compare inner metropolitan to outer metropolitan travel times, at the selected times.

A test of equal proportions was used to compare the number of staff arriving within 30 min during peak hour to those arriving within 30 min at midnight. This method was also used to compare the number of staff arriving at the inner metropolitan hospital within 30 min to the outer metropolitan hospital at selected time points.

All statistical analyses were performed using R statistical environment (13).

## Results

Inner metropolitan staff who were rostered for STEMI recall lived in 17 different postcodes compared to outer metropolitan staff who lived in 22 different postcodes. Therefore, 170 inner metropolitan addresses were randomly generated *via* Google Maps API and 220 addresses for the outer metropolitan postcodes. Results are further summarized in Table 1.

**Table 1:** Summary of results.

		Inner metropolitan	Outer metropolitan	*p*-Value
Number of staff/postcodes		17	22	
Number of random addresses		170	220	

**Travel time**
Maximum (min)	Midnight	29.3	40.9	
	6 p.m.	48.0	57.6	
	7 a.m.	57.0	51.9	

Mean (min)	Midnight	17.0	24.0	
	6 p.m.	30.5	30.0	
	7 a.m.	26.5	27.7	

Mean additional time traveled relative to midnight travel, min ± SE	6 p.m.	13.4 ± 0.3*	6.0 ± 0.3*	<0.001
	7 a.m.	9.5 ± 0.4*	3.6 ± 0.2*	<0.001

Distance (km)	Maximum	24	52	
	Mean	8	26	

Longest distance (km) (travel time, min)	6 p.m.	24 (48.0)	52 (51.6)	
	7 a.m.	24 (57.0)	52 (44.3)	

Longest travel time (min) (distance traveled, km)	6 p.m.	48.0 (24)	57.6 (37)	
	7 a.m.	57.0 (24)	51.9 (37)	

**30 min window**
Percentage of staff arrival within 30 min (%)	Midnight	100	70.9	<0.001
	6 p.m.	45.3*	56.1*	=0.021
	7 a.m.	60.0*	59.1*	=0.529

**Beyond 30 min window**
Mean travel time >30 min (min) (range)	6 p.m.	37.6 (30.3–48.0)	42.5 (30.2–57.6)	
	7 a.m.	38.0 (30.1–57.0)	39.0 (30.4–51.9)	

Mean additional time traveled relative to midnight if traveling >30 min (min) (range)	6 p.m.	16.3 (11.8–21.9)	10.0 (0.7–19.8)	
	7 a.m.	15.3 (8.9–28.3)	5.9 (0.6–12.7)	

*Summarizes the number of postcodes and addresses, staff recall travel time, distance and percentage data at three selected time points of midnight, 6 p.m., and 7 a.m. comparing inner to outer metropolitan variables. p-Value in last column compares inner to outer metropolitan variable*.

*p < 0.001 comparing peak hour variable to midnight variable.

### Time and Distance Estimation

The maximum staff travel time to arrive at the inner metropolitan hospital was 57.0 min at 7 a.m. and to the outer metropolitan hospital was 57.6 min at 6 p.m. Mean travel times were longest at 6 p.m. for both hospitals, 30.5 min for inner metropolitan staff and 30.0 min for outer metropolitan staff. Inner metropolitan staff traveled a statistically significnant mean additional 13.4 min at 6 p.m. and 9.5 min at 7 a.m. relative to midnight travel (*p* < 0.001). This was also true for outer metropolitan staff, however the mean additional time traveled was smaller (Table 1). Inner metropolitan staff traveled a mean 7.4 ± 1.5 min more than outer metropolitan staff for a 6 p.m. STEMI recall (*p* < 0.001). At 7 a.m. inner metropolitan staff traveled a mean 5.9 ± 1.4 min more compared to outer metropolitan staff (*p* < 0.001).

**Graph 1:**
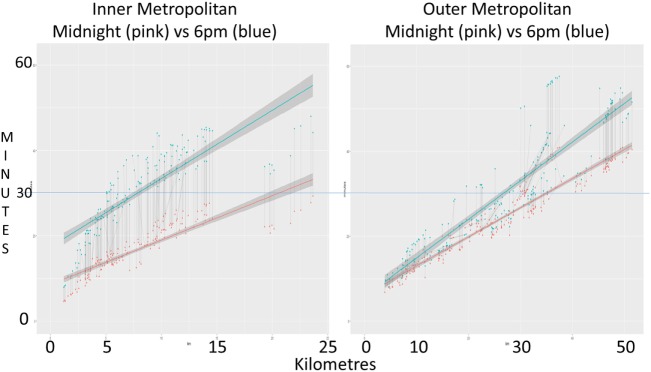
Individual recall travel times. Graph plots recall staff travel time, at midnight (pink) and 6 p.m. (blue), against distance traveled. Both inner (panel 1) and outer metropolitan (panel 2) hospital data are displayed side by side on standardized time (*y*) axis. Line of best-fit shows the general trend. Corresponding dot points are linked by a thin gray line showing the change in individual staff recall travel time from midnight to peak hour.

Graph 1 shows individual recall travel times at 6 p.m. diverge early from midnight travel times and to a greater magnitude over a shorter distance for inner metropolitan staff compared to outer metropolitan staff.

Most outer metropolitan staff traveled a greater distance compared to inner metropolitan staff, mean 26 vs. 8 km (Table 1). Mean distance traveled by staff to reach their destination, comparing midnight to peak hours, for both inner and outer metropolitan hospitals, showed no significant difference. Inner metropolitan staff who traveled the longest time also covered the longest distance. However, for outer metropolitan staff, the longest time traveled was 57.6 min at 6 p.m., corresponding to 37 km traveled, and the longest distance was 52 km taking 51.6 min at 6 p.m.

### Visualization

**Figure 1:**
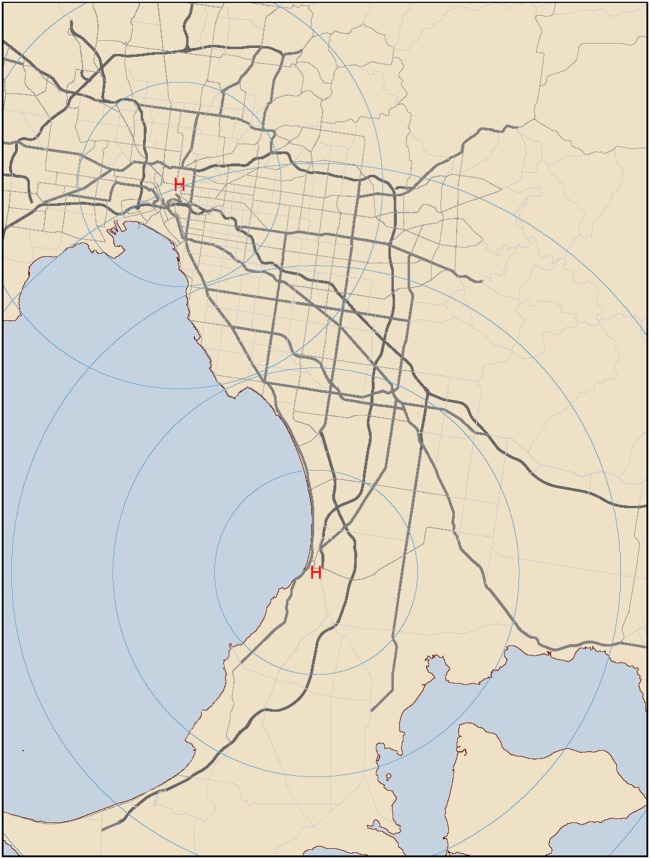
Inner and outer metropolitan Melbourne. Hospitals are depicted by a red letter H. Radial lines around each hospital are spaced at 10 km intervals. Major arterial roads are included. Yellow coloring depicts land, blue depicts water.

Figure 1 shows inner and outer metropolitan Melbourne with the two hospitals depicted by a red letter H. Radial lines around each hospital at spaced at 10 km intervals, to orientate the reading to the Melbourne geography.

**Figure 2:**
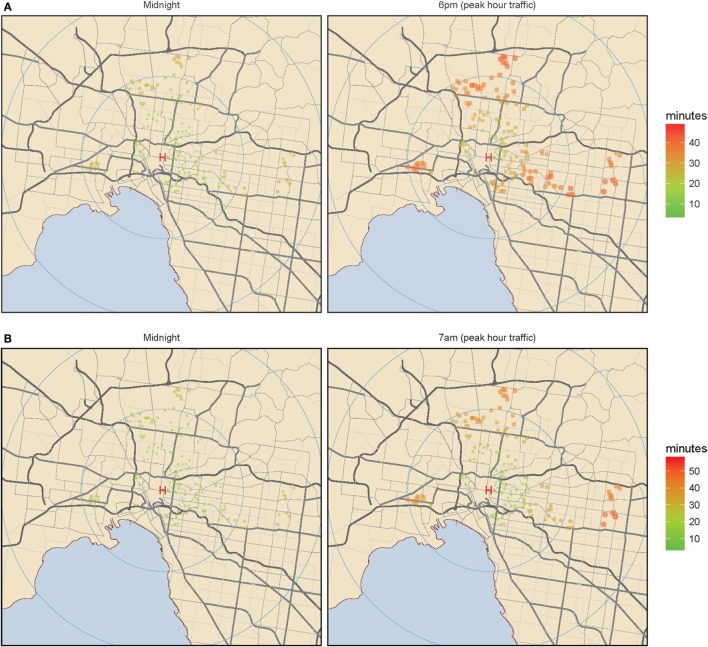
Inner metropolitan Melbourne. **(A)** Midnight color coded staff home addresses according to travel time. Color coded staff home addresses according to travel time. **(B)** Peak hour color coded staff home addresses according to travel time. Color coded staff home addresses according to travel time. Green (shorter time) to red (longer time) color coded travel times and increasing circular diameter represent increasing staff recall travel times for the inner metropolitan hospital, at the three selected time points of midnight, 6 p.m., and 7 a.m. The left panel represents midnight travel and the right panel represents peak hour travel: either 6 p.m. **(A)** or 7 a.m. **(B)**.

Figures 2A,B show peak hour traffic affects inner metropolitan staff recall travel times more if staff live further from the hospital and 6 p.m. recall travel times are worse than at 7 a.m.

**Figure 3:**
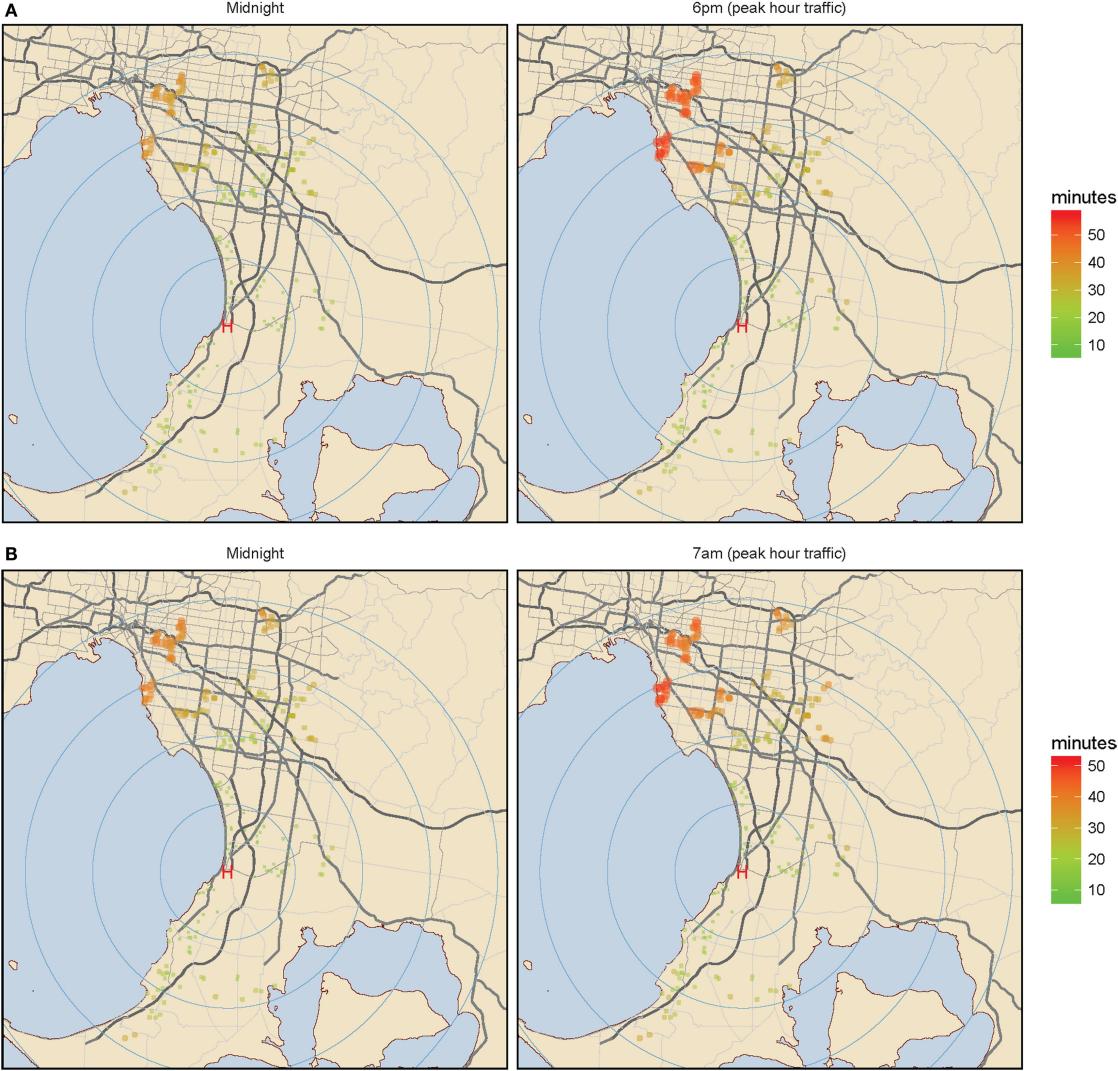
Outer metropolitan Melbourne. **(A)** Midnight color coded staff home addresses according to travel time. Color coded staff home addresses according to travel time. **(B)** Peak hour color coded staff home addresses according to travel time. Color coded staff home addresses according to travel time. Green (shorter time) to red (longer time) color coded travel times and increasing circular diameter represent increasing staff recall travel times for the outer metropolitan hospital, at the three selected time points of midnight, 6 p.m., and 7 a.m. The left panel represents midnight travel and the right panel represents peak hour travel: either 6 p.m. **(A)** or 7 a.m. **(B)**.

Figures 3A,B show peak hour traffic affects outer metropolitan staff recall travel times more if staff live further from the hospital or live in an inner metropolitan location.

### 30 -Min Window

**Graph 2:**
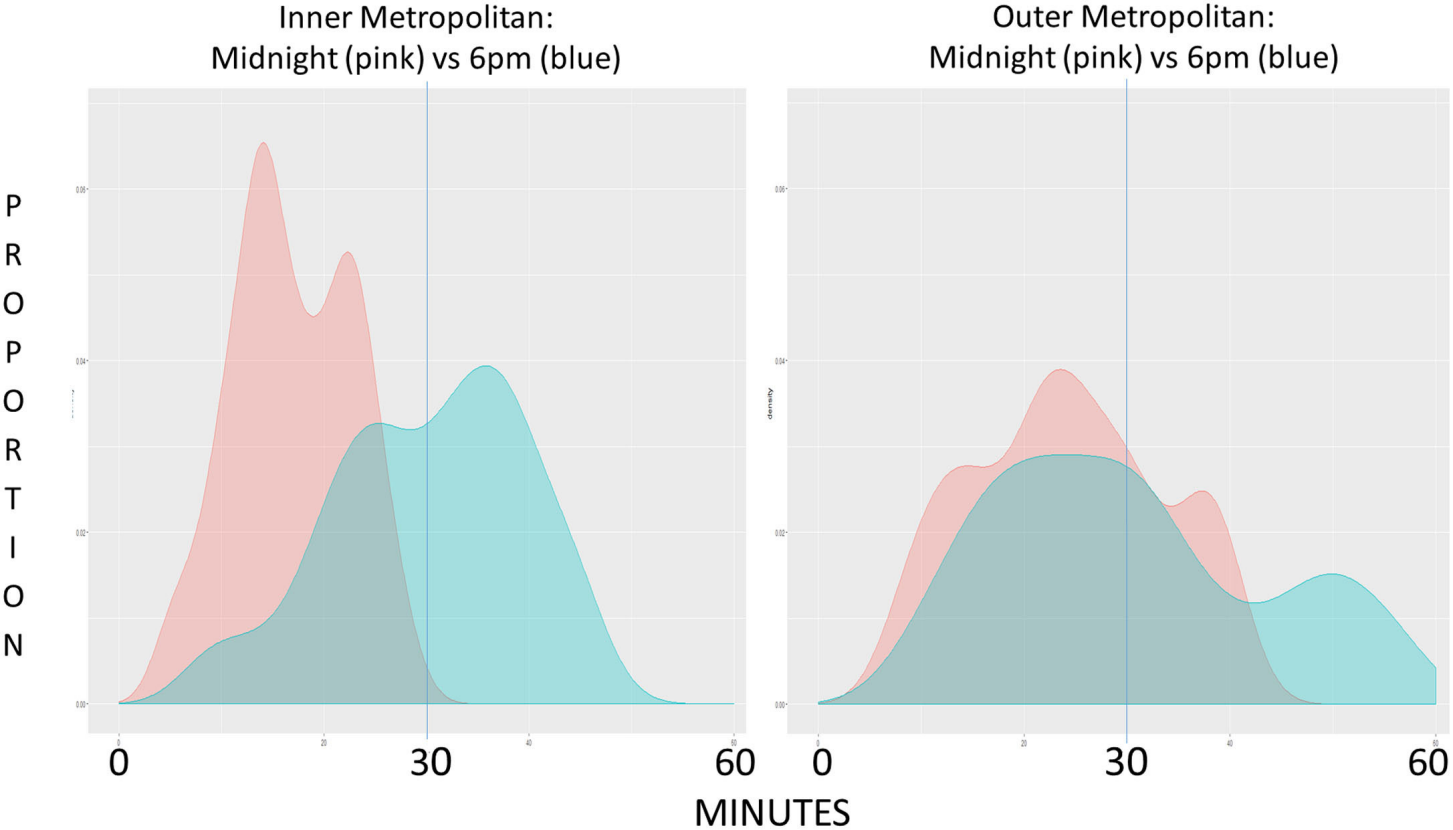
Graph represents the proportion of staff arriving at their destination hospital plotted against time, inner metropolitan (panel 1) and outer metropolitan (panel 2) comparing midnight (pink) to 6 p.m. (blue) data.

Graph 2 shows 100% of inner metropolitan staff traveled less than 30 min at midnight compared to 70.9% of outer metropolitan staff (*p* < 0.001, Table 1). At 6 p.m., less inner metropolitan staff arrived at the hospital within the 30 min window compared to outer metropolitan staff: 45.3 vs. 56.1%, respectively (*p* = 0.021, Table 1). Equal proportions arrive within 30 min at 7 a.m. to the inner and outer metropolitan hospital (60 vs. 59.1%, respectively).

### Beyond 30-Min Window

Mean recall travel times for staff traveling more than 30 min were similar for inner and outer metropolitan hospitals (Table 1). Increases in travel time relative to midnight were 15.3 min for inner metropolitan staff and 5.9 min for outer metropolitan staff at 7 a.m., a difference of 9.4 min between the groups (Table 1).

## Discussion

We have demonstrated, using our novel software, both inner and outer metropolitan catheter laboratory staff recall travel times for STEMI are affected by traffic congestion and distance to destination. Furthermore inner metropolitan staff recall travel times are affected to a greater extent by traffic congestion and outer metropolitan staff recall travels times by distance traveled.

As expected, 6 p.m. and 7 a.m. travel times are significantly longer compared to midnight travel time for both hospitals. Although the maximum and mean recall travel times for inner and outer metropolitan staff were similar during peak hour traffic congestion, these pooled results convey minimal information in regards to the impact of traffic congestion on individual staff. Therefore when analyzed further, the extra time traveled at peak hour compared to midnight became significant and affected the inner metropolitan hospital staff to a greater degree, and was worse during the afternoon peak hour. This is clearly shown on Graph 1 where “lines of best-fit” diverge earlier and to a greater degree for the inner metropolitan staff indicating that the extra time traveled at peak hour is greater and that this change is over a shorter distance traveled compared to outer metropolitan staff.

Inner metropolitan staff traveled a much shorter distance compared to outer metropolitan (mean 8 vs. 24 km, Table 1) explaining the shorter mean midnight travel for inner metropolitan staff compared to outer metropolitan staff: 17 vs. 24 min (Table 1). Mean distance traveled by staff to reach their destination did not significantly change comparing midnight to peak hours for both inner and outer metropolitan hospitals, indicating similar routes were taken at midnight and during peak hours. Importantly some outer metropolitan staff paradoxically took longer to cover a smaller distance and the longest time traveled by outer metropolitan staff members was 57.6 min at 6 p.m., however, these staff only traveled 37 km. This anomaly occurs because these staff members travel from an inner metropolitan home location to the outer metropolitan hospital through heavily congested roads during peak hours.

The proportion of inner metropolitan hospital staff arriving within the stipulated 30-min time frame during peak hour compared to outer metropolitan [45.3 vs. 56.1 at 6 p.m. (*p* = 0.021) and 60.0 vs. 59.1 at 7 a.m. (*p* = 0.529)] clearly demonstrates that inner metropolitan staff are more affected by traffic delay at 6 p.m. (Graph 2). Of concern, only 45.3% of inner metropolitan staff are predicted to arrive within 30 min for a 6 p.m. STEMI recall compared to 56.1% of outer metropolitan staff. Maximum travel times also show that some staff took well over 30 min to arrive at their destination during peak traffic congestion. This is further highlighted by assessing the mean additional time traveled by inner metropolitan staff traveling more than 30 min to arrive at the hospital during peak hour compared to the outer metropolitan group (16.3 vs. 10.0 min at 6 p.m. and 15.3 vs. 5.9 min at 7 a.m.). These findings illustrate that traffic congestion has a far greater impact on staff travel times in areas of high traffic density compared to areas of low traffic density.

Although the main variable analyzed in our study was traffic congestion on time to destination, we were also able to demonstrate the effect of distance on this outcome. We observed that despite the assumption that outer metropolitan hospitals would be less affected by traffic congestion, only 70.9% of staff arrived within 30 min at midnight. This can be explained by the longer mean distance staff traveled to reach the outer metropolitan hospital compared to the inner metropolitan hospital. Overall, we found outer metropolitan staff are more affected by distance traveled than traffic congestion.

With the continued advancement of patient care and improvement in STEMI outcomes, small improvements in minimizing ischemic time become important, with an emphasis on reducing first medical contact to device time. Current guidelines recommend for a ≤90 min time window (3). Historically, focus has been directed at ambulance arrival times and catheter laboratory activation. However, we have found that the environment outside the hospital can have a significant impact on the timely treatment of patients. In this study, we used the example of traffic congestion affecting inner metropolitan staff recall travel time to a greater degree compared to outer metropolitan staff thus showcasing the development and programming of novel computer prediction models. We were able to produce accurate, high-resolution maps representing staff recall travel times, therefore providing staff with a potential resource to help with planning recall travel times. This integration of readily available map software with traffic congestion data to observe variables such as traffic congestion and travel distance, and their impact on staff recall travel time to the hospital for a STEMI is innovative with significant potential. We have previously used similar technology to predict the best combination of hospital hubs providing clot retrieval, ensuring the greatest proportion of patients arriving within 30 min by ambulance for stroke (14). To the best of our knowledge this is the only previous study to utilize this technology to analyze the impact of geographical location as well as traffic congestion on the timeliness of invasive procedures and how it can be used to effectively plan hospital resources.

The delay observed between first medical contact and reperfusion therapy in STEMI requiring coronary intervention is known as “system-delay,” and has significant impacts on patient mortality (10). With our data, it is apparent that staff travel times play an inherent role in system-delay, and acknowledging the impact of this is imperative in order to improve patient care (15). Studies have shown that distance to centers offering primary percutaneous intervention for STEMI has an influence on clinical outcomes and survival (16). Patients who reside further away from invasive centers are also less likely to receive timely angiography and PCI for the treatment of STEMI (17). Although patient distance to PCI centers has been investigated, there is currently a lack of evidence exploring the impact of staff distance to hospital on timely treatment of acute coronary syndromes.

### Limitations

Google Maps API estimated travel time data was not assessed against real travel time data as resource limitations would not have rendered the collection of this information feasible. We have previously demonstrated that the use of optimistic travel time resulted in accurate estimations within 3.5 ± 2.5 min of outer metropolitan real world emergency vehicle travel times (14). However, this does not pertain to staff travel time, and given the time critical nature of STEMI we elected to use the pessimistic traffic model to highlight the worst case scenario.

## Conclusion

We have provided proof-of-concept that novel mapping technology can be used to predict the effect of the environment outside a hospital on the timely treatment of patients. We have shown, as an example of this, that novel mapping technology can predict the impact of both traffic congestion and distance on hospital staff recall travel time. This technology also has broader applications in the provision of other time-dependent medical services as well as addressing other forms of system-delay that contribute to prolonged time to reperfusion in STEMI.

## Author Contributions

All authors meet the editor’s requirements for authorship. RB, TP, and VS have previously published using these concepts and study design (14). All other authors and including RB, TP, and VS have made a substantial contribution to data analysis and interpretation, drafting, revising, and final approval of this manuscript. All authors agree to be accountable for all aspects of the work involved in preparing this manuscript.

## Conflict of Interest Statement

The authors declare that the research was conducted in the absence of any commercial or financial relationships that could be construed as a potential conflict of interest. The reviewer RN and handling editor declared their shared affiliation.

## Footnotes

^1^https://developers.google.com/maps/.

^2^https://www.data.vic.gov.au/data/dataset/postcode-boundaries-polygon-vicmap-admin.
